# Assessing the causal role of adiposity on disordered eating in childhood, adolescence, and adulthood: a Mendelian randomization analysis

**DOI:** 10.3945/ajcn.117.154104

**Published:** 2017-07-26

**Authors:** Zoe E Reed, Nadia Micali, Cynthia M Bulik, George Davey Smith, Kaitlin H Wade

**Affiliations:** 1 Medical Research Council Integrative Epidemiology Unit, School of Social and Community Medicine, Faculty of Health Sciences, University of Bristol, Bristol, United Kingdom;; 2 University College London Institute for Child Health, London, United Kingdom;; 3 Department of Psychiatry, Icahn School of Medicine at Mount Sinai, Mount Sinai, NY;; 4 Departments of Psychiatry and; 5 Nutrition, University of North Carolina at Chapel Hill, Chapel Hill, NC; and; 6 Department of Medical Epidemiology and Biostatistics, Karolinska Institute, Stockholm, Sweden

**Keywords:** ALSPAC, BMI, disordered eating, early life, Mendelian randomization

## Abstract

**Background:** Observational studies have shown that higher body mass index (BMI) is associated with increased risk of developing disordered eating patterns. However, the causal direction of this relation remains ambiguous.

**Objective:** We used Mendelian randomization (MR) to infer the direction of causality between BMI and disordered eating in childhood, adolescence, and adulthood.

**Design:** MR analyses were conducted with a genetic score as an instrumental variable for BMI to assess the causal effect of BMI at age 7 y on disordered eating patterns at age 13 y with the use of data from the Avon Longitudinal Study of Parents and Children (ALSPAC) (*n* = 4473). To examine causality in the reverse direction, MR analyses were used to estimate the effect of the same disordered eating patterns at age 13 y on BMI at age 17 y via a split-sample approach in the ALSPAC. We also investigated the causal direction of the association between BMI and eating disorders (EDs) in adults via a two-sample MR approach and publically available genome-wide association study data.

**Results:** MR results indicated that higher BMI at age 7 y likely causes higher levels of binge eating and overeating, weight and shape concerns, and weight-control behavior patterns in both males and females and food restriction in males at age 13 y. Furthermore, results suggested that higher levels of binge eating and overeating in males at age 13 y likely cause higher BMI at age 17 y. We showed no evidence of causality between BMI and EDs in adulthood in either direction.

**Conclusions:** This study provides evidence to suggest a causal effect of higher BMI in childhood and increased risk of disordered eating at age 13 y. Furthermore, higher levels of binge eating and overeating may cause higher BMI in later life. These results encourage an exploration of the ways to break the causal chain between these complex phenotypes, which could inform and prevent disordered eating problems in adolescence.

## INTRODUCTION

The direction of causation between adiposity and disordered eating in children and adolescents is not well understood. Previous observational studies have shown that higher adiposity, which is usually measured via BMI, is associated with increased risk of developing problematic eating attitudes and eating disorders (EDs) (1–10). For example, a study of 6140 fourteen-year-old males and females from the Avon Longitudinal Study of Parents and Children (ALSPAC) showed that higher BMI at age 10 y was prospectively associated with ED behaviors at age 14 y (9).

However, there has been evidence that reverse causation is also at play, whereby disordered eating in childhood predisposes individuals to excess weight gain in later life (11–18). For example, children with problematic eating attitudes (measured with the use of the Children’s Eating Attitudes Test) at age 11.5 y had 2-fold increased risk of developing new-onset obesity 5 y later in a sample of 13,557 participants from the Promotion of Breastfeeding Intervention Trial (19).

The causal direction of association between adiposity and disordered eating in early life cannot be confidently inferred from observational studies, especially those with a cross-sectional design, because inherent limitations such as confounding, bias, and reverse causation cannot be ruled out. One method that aims to overcome such limitations is an instrumental variable (IV) analysis that is applied within an observational epidemiologic context. For example, Wade et al. (2) used parents’ BMI as an IV to assess the effect of BMI at age 6.5 y on Children’s Eating Attitudes Test scores ≥85th percentile (indicative of problematic eating attitudes) at age 11.5 y in the Promotion of Breastfeeding Intervention Trial. Results suggested that higher BMI in childhood increased risk of developing problematic eating attitudes 5 y later. However, because problematic eating attitudes were not measured any earlier to allow for a formal longitudinal examination of the association, the direction of association was not conclusive.

A well-established application of an IV analysis to further interrogate causality is Mendelian randomization (MR) (20–25). In this application, genetic variants, which are randomly assigned at conception, are used as IVs for an exposure of interest to estimate causal relations between an exposure and outcome in attempts to overcome the issue of confounding and reverse causation. In an extension, two-sample MR enables the gene-exposure and gene-outcome estimates to be generated from 2 independent and larger samples, which are used to estimate the causal effect of the exposure-outcome association with increased statistical power (26).

The aim of the current study was to use MR to infer the causal direction of the association between BMI (in kg/m^2^) and disordered eating in childhood, adolescence, and adulthood. First, we assessed the causal effect of BMI at age 7 y on disordered eating patterns at age 13 y. Second, we estimated the causal effect of the same disordered eating patterns at age 13 y on BMI at age 17 y in the ALSPAC. Third, we conducted a two-sample MR analysis with publically available data to assess the causal direction of the association between BMI and EDs [specifically, anorexia nervosa (AN) and bulimia nervosa (BN)] in adulthood.

## METHODS

### ALSPAC

The ALSPAC cohort is a prospective birth cohort study in which 14,541 pregnant women who were living in Avon, United Kingdom, with an expected delivery date from 1 April 1991 to 31 December 1992 were enrolled (27). Of these deliveries, 13,988 children were still alive 1 y later and have been followed up with regular questionnaires and clinical measures that provided behavioral, lifestyle, and biological data. The study website contains details of all the data that are available through a fully searchable data dictionary (http://www.bristol.ac.uk/alspac/researchers/access/). Participants who had withdrawn consent or who were related (only first-born children were included) were excluded from the study for the current analyses (*n* = 122).

### Ethics

Ethical approval for the study was obtained from the ALSPAC Ethics and Law Committee and the local research ethics committees.

### Measures of BMI

When children were 7 y old, they were invited to attend the research clinic, and a total of 8297 children attended. Height was measured to the nearest millimeter with the use of a Harpenden Stadiometer (Holtain Ltd.). Weight was measured via the Tanita Body Fat Analyzer (Tanita TBF UK Ltd.) to the nearest 50 g. BMI was calculated by dividing weight by height squared. Weight and height measures were collected, and BMI was calculated according to the same methods as were used at the research clinics at ages 13 y (*n* = 6832) and 17 y (*n* = 5217) with weight recorded to the nearest 0.1 kg at age 13 y.

### Disordered eating patterns

When the children were 13 y old, parents of 10,135 children (and an additional 479 children in a second phase) were sent a questionnaire version of the ED section of the Developmental and Well-being Assessment (28), which is a semistructured interview for psychiatric diagnoses in children and adolescents. A total of 7165 questionnaires were returned. The section consisted of 28 questions, which provide information about ED behaviors and cognitions, impairment on the adolescent’s life as a result of the ED symptoms, and burden on the family and parents as a result of the ED symptoms. The cognitions and behaviors that were investigated included a fear of weight gain, being upset or distressed about weight and shape, the avoidance of fattening foods, food restriction, exercising for weight loss, and purging behaviors. Of these questions, 16 questions (15 questions in males) were used in previous exploratory structural equation models that derived 3 latent dimensions as follows: *1*) binge eating and overeating, *2*) weight or shape concern and weight-control behaviors (WCBs), and *3*) food restriction (17). The disordered eating patterns can be interpreted on the SD scale, with higher scores indicating greater disordered eating.

### Covariates

Data for the child’s age at the time of measuring the outcome, sex, infant birth weight, gestational age, and recent measures of diet and physical activity plus mother’s prepregnancy BMI and socioeconomic status were included as covariates. Infant birth weight was recorded at birth or from birth notification, and gestational age was estimated from clinical records (29). Prepregnancy weight was predicted from multilevel models on the basis of obstetric medical records, and height was based on maternal report (30). Mother’s prepregnancy BMI was calculated as previously described for children. Socioeconomic status was derived from the highest social class on the basis of the occupations of the mother and partner from questionnaire data at ∼32 wk of gestation.

Dietary intake likely confounds the association between BMI at age 7 y and the disordered eating patterns at age 13 y (6, 11). Therefore, we included total caloric intake at age 6 y as a covariate. Total caloric intake at age 6 y was previously estimated with linear-spline multilevel models of diet diaries and food-frequency questionnaires (31). Similarly, total caloric intake at age 12 y was used as a covariate in the association between disordered eating patterns at age 13 y and BMI at age 17 y.

Physical activity was measured with the use of an MTI Actigraph AM7164 2.2 accelerometer (Actigraph; www.theactigraph.com), which was worn for 7 d at the 11-y clinic. Of the 6622 children who agreed to wear the accelerometer, 5595 children had valid physical activity data. Mean daily physical activity [counts per minute (CPM)] and mean moderate-to-vigorous intensity physical activity (defined as being >3600 CPM) were used as covariates in the association between disordered eating patterns at age 13 y and BMI at age 17 y (32).

### Genetic variation

Genotyping was conducted for children in the ALSPAC with the use of the Illumina HumanHap550 quad chip genotyping platform. Genome-wide data were generated by Sample Logistics and Genotyping Facilities at the Wellcome Trust Sanger Institute and LabCorp (Laboratory Corporation of America) with the use of support from 23andMe, a privately held personal genomics and biotechnology company. Data were subject to quality-control measures, and individuals were excluded because of having ≥1 sex mismatches, minimal or excessive heterozygosity, disproportionate missingness, or insufficient sample replication. A total of 9115 children and 500,527 single nucleotide polymorphisms (SNPs) passed the filters. Imputation of the data was performed with the use of a phased version of the 1000 Genomes reference panel from the Impute2 reference data repository. After further quality control, imputation and the removal of subjects who had withdrawn consent, there were 8252 children with full genotype data available.

### Statistics

#### Observational associations

Observational associations between BMI at age 7 y and disordered eating patterns at age 13 y were assessed with multivariable linear regression in 3 models as follows: *1*) adjusted for age at which disordered eating was assessed; *2*) adjusted for age of disordered eating measure, child’s birth weight, gestational age, mother’s prepregnancy BMI, and socioeconomic status; and *3*) further adjusted for total caloric intake at age 6 y. Because the prevalence and presentation of disordered eating are known to vary between males and females (2, 9, 14, 33, 34), analyses were stratified by sex.

#### MR analyses

**FIGURE 1:**
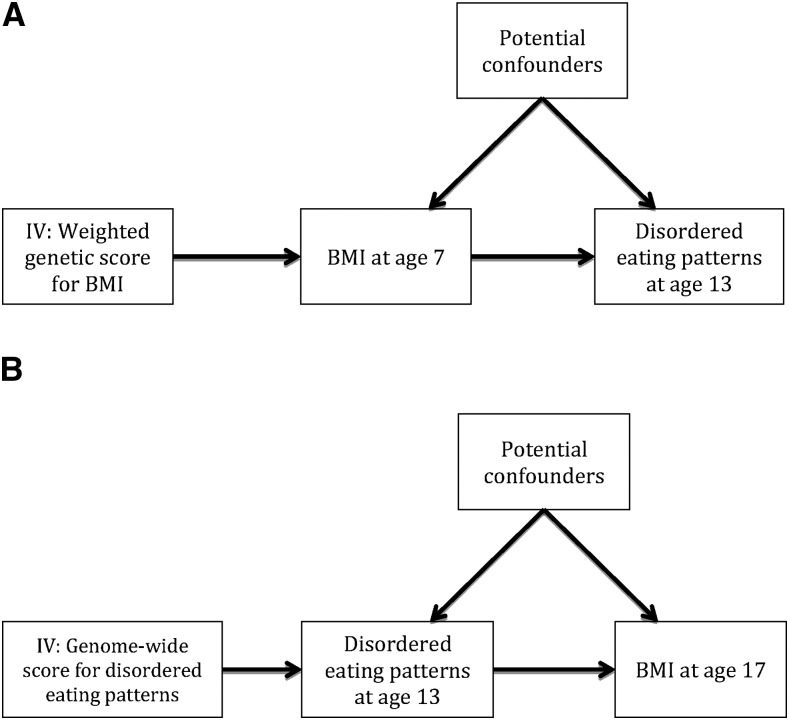
Mendelian randomization applied to investigating the causal direction of the association between BMI and disordered eating. Age is expressed in y. (A) DAG for the causal effect of higher BMI causing disordered eating whereby a weighted allelic score is used as an IV for BMI. (B) DAG for the causal effect of disordered eating causing variation in BMI whereby a genome-wide prediction score is used as an IV for disordered eating patterns. DAG, directed acyclic graph; IV, instrumental variable.

A genetic score for BMI was constructed in the ALSPAC with the use of external weightings from published data from the Genome-Wide Investigation of Anthropometric Traits (GIANT) consortium, which included ≤339,224 individuals for BMI (35). A total of 96 independent SNPs were available in the ALSPAC genetic data. Similar to a previous methodology (36), a weighted genetic score for BMI was generated by multiplying the dose of the effect allele at each SNP (0, 1, or 2), which was extracted with the use of QCTOOL v1.4, by the effect estimate on each SNP on BMI from the genome-wide association study (GWAS) and taking the sum of these values. These values were divided by the total sum of the effect estimates from the GWAS and subsequently multiplied by the total number of SNPs influencing that trait. Therefore, the genetic score reflects the number of average BMI-increasing alleles that are carried by each individual. The resulting weighted genetic score was used in the 2-stage least-squares MR analyses as an IV for BMI to improve the causal inference in the association between BMI at age 7 y and disordered eating patterns at age 13 y (**Figure 1A**).

#### Sensitivity analyses

A sensitivity analysis was conducted with the use of the inverse-variance weighted (IVW) and MR-Egger methodologies, which are distinct methodologies that aim to provide estimates of a causal effect of the exposure (here, BMI) on an outcome of interest (here, disordered eating patterns) with varying assumptions of instrument validity. The IVW approach assesses the causal exposure-outcome association, constraining the intercept term to zero, and, therefore, assumes all variants are valid IVs with no pleiotropic effect on the outcome (i.e., no effect of any SNP on the outcome independent of the exposure). One indication of pleiotropy is a high degree of heterogeneity between causal estimates of the individual SNPs combined with the use of the IVW method. Therefore, Cochran’s test was used in the IVW method to test for evidence of heterogeneity between individual SNPs. The MR-Egger approach (unlike the IVW method) allows the intercept term (of the causal exposure-outcome association) to vary. The value of this intercept can be interpreted as an estimate of the overall directional pleiotropy across the genetic variants with a nonzero intercept term indicating directional pleiotropy (thus, invalidating MR assumptions). The slope of the MR-Egger test provides an estimate of the causal exposure-outcome association accommodating any pleiotropic effects (37). For the MR-Egger method, Rucker’s *Q* test was used to assess the heterogeneity between individual SNPs while adjusting for directional pleiotropy (38). The weighted median approach was also conducted as a sensitivity analysis, which allows ≤50% of the SNPs that are used to be invalid (i.e., pleiotropic) (39).

Second, as a sensitivity analysis to assess the causal direction of the association between BMI and disordered eating patterns, we conducted both observational and MR analyses of the association between disordered eating patterns at age 13 y and BMI at age 17 y in the ALSPAC sample. The observational association between disordered eating patterns at 13 y of age and BMI at age 17 y was assessed via multivariable linear regression with the use of the same 2 models as described previously with an additional model adjusting for BMI that was measured at the age of 13 y.

Because, to our knowledge, there have been no GWASs that have been conducted for the disordered eating patterns, we conducted a split-sample GWAS to obtain genome-wide prediction scores to use as IVs for the disordered eating patterns at age 13 y in MR analyses (Figure 1B). To do this, the total sample was randomly split into 2 subsamples, and genome-wide prediction scores for the 3 disordered eating patterns were generated for each separate subsample. The genome-wide significant SNPs that were associated with BMI (from the GIANT study) were excluded to avoid direct pleiotropy. These scores were used as IVs for the disordered eating patterns in the other independent subsample in a 2-stage least-squares MR analysis that assessed the effect of disordered eating patterns at age 13 y on BMI at age 17 y. This method has previously been used to assess the causal direction of the association between physical activity and BMI (32). Finally, for the genetic scores that were used as IVs for both BMI and the disordered eating patterns, associations with the confounders were tested, and any that were strongly associated were further added as covariates in MR analyses as a sensitivity analysis. All analyses were conducted with the use of Stata software (version 14; StataCorp) and R 3.2.0 software (R Core Team).

#### Two-sample MR analyses

A two-sample MR approach (40) was conducted to assess the causal direction of the association between BMI and disordered eating (specifically, EDs) in adults with the use of publically available data from the GIANT consortium and the Wellcome Trust Case Control Consortium (WTCCC3) (*n* = 17,767 with 2907 cases). To assess the causal effect of BMI on AN, estimates of the association between the 97 BMI SNPs from the GIANT GWAS (35) and AN were obtained from the WTCCC3 data set (41). Similarly, the causal effect estimate of AN on BMI was calculated by obtaining estimates for the association between the 15 AN SNPs from the WTCCC3 GWAS (41) and BMI from the GIANT data set. To generate a causal estimate of the effect of BMI on AN (and the reverse), the SNP-exposure and SNP-outcome effect estimates were combined with the use of IVW, weighted median, and MR-Egger methodologies, as described previously. The online MR-Base tool was used to conduct two-sample MR analyses with appropriate harmonization and merging of the exposure and outcome GWAS summary-level data (42). In the same way, we also conducted analyses to assess the association between BMI and BN with the use of the GIANT GWAS (35) and the 23 SNPs that were most associated with BN from Wade et al. (43).

## RESULTS

### Cohort description

Full data on the exposure and outcome (BMI at age 7 y and disordered eating patterns at age 13 y, respectively) were available for 5780 participants. Of these participants, genetic data were available for 4473 individuals. Descriptive statistics for the variables that were used are presented in **Table 1**.

**TABLE 1:** Description of cohort for variables used in analyses^1^

Variable	*n*^2^	Value
Baseline measures		
Sex, F, %	19,254	48.40
Gestational age, wk	14,402	38.45 ± 5.48^3^
Birth weight, g	13,699	3392.89 ± 570.23
Mother’s prepregnancy BMI, kg/m^2^	11,516	22.93 ± 3.83
Highest socioeconomic status of mother and partner, % nonmanual	11,824	52.82
BMI measures		
Age at 7-y clinic	6616	7.54 ± 0.32
BMI at age 7 y, kg/m^2^	6616	16.27 ± 2.11
Age at 17-y clinic	5008	17.81 ± 0.44
BMI at age 17 y, kg/m^2^	5008	22.87 ± 4.24
Disordered eating measures		
Age when disordered eating data collected, y	7076	13.16 ± 0.18
Binge eating and overeating pattern, SD		
Male	2577	−0.31 ± 0.77
Female	2989	−0.08 ± 0.76
Weight and shape concerns and WCBs, SD		
Male	2577	0.05 ± 0.84
Female	2989	0.02 ± 0.87
Food restriction, SD		
Male	2577	−0.15 ± 0.68
Female	2989	0.35 ± 0.62
Dietary and physical activity measures		
Predicted caloric intake at age 6 y	11,882	1555.46 ± 128.73
CPM at age 11 y	5876	605.72 ± 181.97
MVPA at age 11 y, min	5876	23.06 ± 15.49

1 CPM, counts per minute; MVPA, moderate-to-vigorous intensity physical activity; WCB, weight control behavior.

2 Data for each of the variables were available after the removal of individuals who were related or had withdrawn consent.

3 Mean ± SD (all such values).

### Observational associations

After adjusting for confounders, there was a positive association between BMI at age 7 y and both binge eating and overeating in both males [difference in binge eating and overeating score per unit (kg/m^2^) increase in BMI: 0.19 SDs (95% CI: 0.17, 0.21 SDs); *P* = 3.96 × 10^−82^] and females [difference: 0.13 SDs (95% CI: 0.11, 0.14 SDs); *P* = 5.25 × 10^−50^] and weight and shape concerns and WCBs for males [difference: 0.25 SDs (95% CI: 0.23, 0.27 SDs); *P* = 4.65 × 10^−123^] and females [difference: 0.20 SDs (95% CI: 0.19, 0.22 SDs); *P* = 2.13 × 10^−104^] at age 13 y. There was also a positive association with food restriction in males at age 13 y [difference: 0.13 SD (95% CI: 0.11, 0.14); *P* = 1.65 × 10^−41^], which was much smaller in females [difference: 0.03 SDs (95% CI: 0.02, 0.05 SDs); *P* = 1.63 × 10^−5^] (**Table 2**). There was evidence of sex-differences for all disordered eating patterns with consistently larger estimates in males than in females (**Table 3**). However, the estimates in males generally had wider 95% CIs.

**TABLE 2:** Results for the observational and MR analyses of the association between BMI at age 7 y and disordered eating patterns at age 13 y^1^

		Observational analyses								
		Adjusted for age		Adjusted for age and confounding factors^3^		Adjusted for all confounders and diet		MR analyses		
Disordered eating pattern	*n*^2^	β (95% CI)^4^	*P*	β (95% CI)^4^	*P*	β (95% CI)^4^	*P*	β (95% CI)^4^	*P*	Wu-Hausman *P*^5^
Male										
Binge eating and overeating	1582	0.21 (0.19, 0.22)	1.46 × 10^−141^	0.20 (0.18, 0.21)	2.38 × 10^−109^	0.19 (0.17, 0.21)	3.96 × 10^−82^	0.30 (0.20, 0.40)	9.17 × 10^−9^	0.10
Weight and shape concerns and WCBs	1582	0.26 (0.25, 0.28)	7.30 × 10^−197^	0.26 (0.24, 0.28)	6.94 × 10^−162^	0.25 (0.23, 0.27)	4.65 × 10^−123^	0.39 (0.29, 0.50)	1.40 × 10^−12^	0.01
Food restriction	1582	0.12 (0.10, 0.13)	1.93 × 10^−55^	0.12 (0.10, 0.13)	7.00 × 10^−46^	0.13 (0.11, 0.14)	1.65 × 10^−41^	0.09 (0.0004, 0.19)	0.05	0.57
Female										
Binge eating and overeating	1763	0.14 (0.13, 0.15)	5.97 × 10^−92^	0.13 (0.12, 0.15)	5.37 × 10^−70^	0.13 (0.11, 0.14)	5.25 × 10^−50^	0.17 (0.09, 0.25)	4.98 × 10^−5^	0.58
Weight and shape concerns and WCBs	1763	0.22 (0.20, 0.23)	5.37 × 10^−177^	0.21 (0.20, 0.23)	6.56 × 10^−140^	0.20 (0.19, 0.22)	2.13 × 10^−104^	0.32 (0.24, 0.41)	1.07 × 10^−12^	0.01
Food restriction	1763	0.02 (0.01, 0.04)	2.60 × 10^−5^	0.03 (0.01, 0.04)	5.13 × 10^−5^	0.03 (0.02, 0.05)	1.63 × 10^−5^	0.005 (−0.07, 0.08)	0.90	0.65

1 MR, Mendelian randomization; WCB, weight control behavior.

2 Number of individuals included in the fully adjusted model who also had genetic data available.

3 Adjusted for gestational age, birth weight, socioeconomic status, and maternal prepregnancy BMI.

4 Effect estimates represent the change in each disordered eating pattern (SD) per unit increase in BMI (in kg/m^2^) at age 7 y.

5 *P* values for the comparison of observational results with MR results.

**TABLE 3:** Results of the interaction term (BMI by sex) for the fully adjusted observational analyses for males and females combined^1^

Disordered eating pattern	*n*	β (95% CI)^2^	*P*
Binge eating and overeating	4223	−0.06 (−0.08, −0.04)	8.11 × 10^−9^
Weight and shape concerns and WCBs	4223	−0.05 (−0.07, −0.02)	3.66 × 10^−5^
Food restriction	4223	−0.10 (−0.11, −0.08)	4.27 × 10^−22^

1 Effect estimates represent the change in each disordered eating pattern (SD) per unit increase in the interaction term (i.e., for the comparison of females with males). WCB, weight control behavior.

2 Adjusted for all covariates, sex, and BMI-by-sex interaction term.

### Association between the BMI genetic score and BMI at age 7 y

The genetic score was associated with BMI at age 7 y [difference in mean BMI per allele increase: 0.06 (95% CI: 0.05, 0.07); *P* = 2.39 × 10^−33^] and explained 3% of the variance (**Table 4**).

**TABLE 4:** Association between BMI genetic score and BMI at age 7 y^1^

	*n*	β (95% CI)^2^	*P*	*R*^2^
BMI at age 7 y	4473	0.06 (0.05, 0.07)	2.39 × 10^−33^	0.03

1 Effect estimates represent the change in BMI (in kg/m^2^) per allele increase in the genetic risk score. *R*^2^ represents the variance explained in BMI by the weighted genetic score.

2 Adjusted for age and sex.

### MR analyses

MR analyses indicated that higher BMI at age 7 y had a likely causal effect on higher levels of binge eating and overeating in males [difference in binge eating and overeating score per unit increase in BMI: 0.30 SDs (95% CI: 0.20, 0.40 SDs); *P* = 9.17 × 10^−9^] and females [difference: 0.17 SDs (95% CI: 0.09, 0.25 SDs); *P* = 4.98 × 10^−5^] and weight and shape concerns and WCBs in males [difference: 0.39 SDs (95% CI: 0.29, 0.50 SDs); *P* = 1.40 × 10^−12^] and females [difference: 0.32 SDs (95% CI: 0.24, 0.41 SDs); *P* = 1.07 × 10^−12^] at age 13 y. There was evidence for a weaker causal effect of higher BMI at age 7 y on higher levels of food restriction in males at age 13 y [difference: 0.09 SDs (95% CI: 0.0004, 0.19 SDs); *P* = 0.05], and this effect was much weaker in females (Table 2). The effect estimates from the MR analyses were larger than those from the observational analyses for weight and shape concerns in both males and females (*P* = 0.01; Wu-Hausman test).

### Sensitivity analyses

There was no evidence of a difference between the main and sensitivity analyses with the use of IVW and MR-Egger approaches (*P* = 0.23–0.83) (**Supplemental Table 1**). There was also no evidence of heterogeneity between the causal estimates that were obtained with the use of each SNP in the IVW analyses (*P* = 0.27–0.997) even when accounting for directional pleiotropy (*P* = 0.25–0.997) (Supplemental Table 1, **Supplemental Figure 1**). There was varying evidence of an effect of pleiotropy (*P* = 0.16–0.83), which slightly attenuated the effect estimate for binge eating and overeating and food restriction in males with rs7899106 and rs13201877 for binge eating and overeating and rs11847697 for food restriction potentially being the main drivers of any pleiotropic effect. Finally, the weighted median approach revealed similar causal effects to those of the IVW and MR-Egger methods (Supplemental Table 1), thereby indicating that, even under the assumption that 50% of the IVs were invalid, the effect estimates were not substantially changed.

Maternal prepregnancy BMI and predicted caloric intake at age 6 y were associated with the genetic score for BMI (**Supplemental Table 2**), and adjusting for these variables in the MR analyses (**Supplemental Table 3**) made little difference to the results. However, the finding for food restriction in males was attenuated.

When we assessed the causal effect of disordered eating patterns at age 13 y and BMI at age 17 y, there was a positive observational association of binge eating and overeating at age 13 y and BMI at age 17 y in males [difference in mean BMI per SD increase in the binge eating and overeating score: 2.10 (95% CI: 1.86, 2.34); *P* = 7.82 × 10^−58^] and females [difference: 1.90 (95% CI: 1.67, 2.13); *P* = 1.07 × 10^−54^] and weight and shape concerns and WCBs at age 13 y and BMI at age 17 y in males [difference: 2.16 (95% CI: 1.94, 2.38); *P* = 3.73 × 10^−70^] and females [difference: 1.83 (95% CI: 1.63, 2.03); *P* = 6.09 × 10^−67^]. There was also a positive association between food restriction in males and BMI [difference: 1.22 (95% CI: 0.92, 1.51); *P* = 1.01 × 10^−15^], which was much smaller in females [difference: 0.42 (95% CI: 0.12, 0.72); *P* = 0.006] (**Supplemental Table 4**). The addition of BMI at age 13 y to the model attenuated the effect sizes for these associations toward the null (Supplemental Table 4) with a positive (albeit weaker) association of binge eating and overeating and BMI in both males and females still being observed.

In split-sample analyses, the genome-wide prediction scores that were generated for binge eating and overeating in males and weight and shape concerns and WCBs in both males and females were associated with the corresponding disordered eating patterns (**Supplemental Table 5**). In MR analyses using these genome-wide prediction scores, there was evidence of a causal effect of higher levels of binge eating and overeating in males at age 13 y and higher BMI at age 17 y [difference in mean BMI per SD increase in the binge eating and overeating score: 4.11 (95% CI: 0.25, 7.96); *P* = 0.04]. There was no evidence to suggest a causal effect of any other disordered eating pattern at age 13 y on BMI at age 17 y (**Supplemental Table 6**). Sensitivity analyses showed that maternal prepregnancy BMI, CPM at age 11 y, moderate-to-vigorous intensity physical activity at age 11 y, and BMI at age 13 y were associated with the genome-wide prediction score for weight and shape concerns and WCBs in females at age 13 y (**Supplemental Table 7**), and adjustment for these variables in the MR analysis reversed the direction of the effect; however, 95% CIs were wide (**Supplemental Table 8**).

### Two-sample MR analyses

Two-sample MR analyses were conducted to investigate the relation between BMI and EDs (AN and BN) in adults. After harmonization and merging of the GWAS summary-level data, there were 74 and 88 BMI SNPs that were available to estimate the causal effect of BMI on AN and BN, respectively. For the reverse direction, there were 8 AN SNPs and 10 BN SNPs that were available to estimate the causal effect of AN and BN on BMI after harmonization and merging of the GWAS summary-level data. There was no indication of a causal effect of higher BMI and AN (**Supplemental Table 9**) or the reverse (**Supplemental Table 10**) or of a causal effect of higher BMI on BN (**Supplemental Table 11**) or the reverse (**Supplemental Table 12**).

## DISCUSSION

Within this study, we showed evidence of a causal effect of higher BMI at age 7 y on higher levels of binge eating and overeating, weight and shape concerns, and WCBs in both males and females and food restriction in males at age 13 y. Furthermore, MR results also suggested a likely causal effect of binge eating and overeating at age 13 y on higher BMI at age 17 y in males with a considerable ∼4.0-higher BMI with each SD increase in the disordered eating score. This is the first study, to our knowledge, to use genetic scores as IVs in an MR approach to provide evidence for this causal relation.

Building on the current literature, these results support the causal effect of elevated BMI in childhood and disordered eating later in life (1–3, 5–9). In this context, individuals with higher BMI in early life may be at higher risk of developing disordered eating patterns (2, 7). Overweight children, as early as 5 y old, have reported behaviors such as lower body esteem and self-esteem than have thinner children because overweight children are likely to be subject to social pressures that support thinner body shapes (2, 44, 45).

In addition, our results also suggest that the reverse association may be true (i.e., that binge eating and overeating in adolescence likely causes higher BMI at age 17 y), which may be a direct result of loss-of-control eating and binge eating on weight (11, 12). Individuals with a variety of disordered eating patterns (weight control and dietary restraint, similarly to those included in the current study) in early life are likely to gain more weight than are those without such behaviors (11, 13, 14). Therefore, large studies with the ability to explicitly test the causal effect of a range of disordered eating patterns and attitudes on later adiposity, as was done in this current study, would be beneficial to further understand the mechanisms that link these 2 complex phenotypes.

A previous study that used the ALSPAC data set showed that a BMI polygenic risk score (and the fat mass and obesity-associated gene polymorphism alone) was associated with binge eating later in adolescence, thereby suggesting either that the 2 phenotypes may share some genetic etiology or that these traits may be causally linked (46). In the current study, there was no strong evidence of pleiotropic effects, in either direction, with any particular SNP (i.e., no effect of the genetic variants on the outcome of interest independent of the exposure). Therefore, the previously reported association between the BMI polygenic risk score and binge eating behavior may reflect causality.

Our results also highlight the sex differences in the relation between BMI and disordered eating patterns. However, compared with other literature that has investigated BMI and such patterns (2, 8, 47), this study indicated stronger relations in males. One study showed that weight concern and dieting were more strongly associated with BMI in males than females (48). However, authors have suggested that this result may be due to the lower frequency of weight concern and dieting in males who have lower BMI. Therefore, participants who exhibited the disordered behaviors may generally have higher BMI.

Our analyses did not provide evidence of a causal relation between BMI and EDs (in either direction) in adults, which could reflect the absence of causality at this stage in life. However, because of the relatively small sample size of the AN GWAS (*n* = 2907 cases) and the very small number of BN cases in Wade et al. (43) (*n* = 151) that were used here, alongside the absence of genome-wide significant SNPs associated with AN and BN (e.g., none of the 23 SNPs from the BN GWAS reached genome-wide significance, and they were not replicated), there was a lack of power (and weak instrument bias) within these analyses. However, the direction of the effect between BMI and AN is consistent with previous research, which indicates an inverse SNP-based genetic correlation between AN and BMI, extreme BMI, being overweight, and the body fat percentage (49, 50). Although we showed no evidence to support causality in this context, the inverse genetic correlation between EDs and BMI may support our initial findings, suggesting that binge eating specifically, as opposed to AN (more drastic measures of weight control such as fasting, skipping meals, and vomiting) (51), is more likely causally linked to higher BMI. However, it is important to consider the phenotypic differences being studied between the younger and adult populations within the current study (i.e., disordered eating patterns compared with frank EDs); therefore, the results across different stages of the life course are not directly comparable.

The strengths of this study are the large sample size and the availability of prospectively collected data at different ages that were obtained from the ALSPAC cohort. In addition, the use of MR can overcome inherent limitations of observational epidemiologic studies because the general assumptions are met. First, the IV that was used for BMI was strongly associated with BMI in this sample, and the IV for the disordered eating patterns were also associated with the corresponding exposures, thus satisfying the first assumption of the MR methodology. Second, the use of genetic variants as instruments overcomes the issue of confounding and reverse causation because these variants are randomly assigned at conception and remain fixed throughout the life course. Third, measurement error is reduced as a result of the use of genotype data, which is measured to a high degree of accuracy. In addition, we showed that the IV is independent of potentially confounding factors by checking these associations directly, and sensitivity analyses that adjusted for these made little difference to the results. Another assumption of MR is that the IV is independent of the outcome because of the exposure and factors that confound the exposure-outcome relation (horizontal pleiotropy). With the novel methods (to our knowledge) such as MR-Egger and weighted median approaches used here, we were able to directly test for heterogeneity and pleiotropic effects within our analyses and showed little evidence of either.

However, there are some limitations to this study. First, to our knowledge, there have been no GWASs of the disordered eating patterns that were used; therefore, we were unable to perform MR to assess the causal effect of disordered eating patterns at age 13 y on BMI at age 17 y in the conventional manner. Although we used a split-sample technique to overcome this matter, there is an issue of decreased power with this method whereby estimates tend to be more biased toward the null (52). Furthermore, because of the genome-wide nature of the prediction score and thresholds that were used, this approach is generally more likely to be subject to pleiotropy and confounding as some of the variants that were included in the IV for the disordered eating patterns may also be associated indirectly with BMI. However, to limit the possible pleiotropic effect of SNPs that were included in the IV that was generated for the disordered eating patterns to the best of our ability, we removed SNPs that are known to be associated with BMI.

The genetic score for BMI and the genome-wide prediction scores for some of the disordered eating patterns are associated with some of the considered confounders, thereby invalidating one of the MR assumptions. However, adjusting for these factors did not change the results substantially. Furthermore, because maternal BMI (one of the confounders that is associated with the BMI genetic score) shares common genetic variation with the child’s BMI, the associations that were shown were not unexpected. Last, MR has some other general limitations such as canalization and population stratification, the latter of which is unlikely to have had a large impact on our results because related individuals were removed, and the sample that was used is of European ancestry.

In conclusion, our results suggest that higher BMI in childhood is likely to cause disordered eating in adolescence. Furthermore, our results suggest that there is evidence of a causal effect of binge eating and overeating in males during early adolescence and higher BMI 5 y later. However, further work is needed to replicate these findings, especially after larger GWAS of disordered eating patterns and EDs emerge. These results encourage the exploration of ways in which to break the causal chain between childhood BMI and later disordered eating patterns (and for the link between binge eating and overeating with later higher BMI). Such research could inform and prevent the emergence of disordered eating in adolescence and may, therefore, reduce the incidence of such behaviors and associated metabolic disturbances later in life.
